# Supplementary material for: COVID-19 Phenotypes and Comorbidity: A Data-Driven, Pattern Recognition Approach Using National Representative Data from the United States
Source: Int J Environ Res Public Health. 2022 Apr 12;19(8):4630. doi: 10.3390/ijerph19084630 (PMC9029400; doi:10.3390/ijerph19084630)
Supplement: Supplementary file 1 [file ijerph-19-04630-s001.zip › ijerph-1645495-supplementary S1-S5.pdf]

Supplementary Materials

Logistic probability case scoring

The logistic regression model extracted from August's data was subsequently used to score subsequent cases per month. Let us consider a model where x_1,x_2,…,x_n are symptoms (used as predictors) of the binary response variable C (1=COVID-19 positive, 0=COVID-19 negative), which represents COVID-19 status. The log-odds λ of the probability p of C=1 can be presented as follows:

$$\lambda = ln\left(\frac{p}{1-p}\right) = b_0 + b_1x_1 + b_2x_2 + \cdots b_nx_n, \qquad 0 < p < 1 \qquad \text{(S1)}$$

where b_1,b_2,…,b_n are the model parameters, b_0 is the constant and 1–p is the probability of C=0. The exponent of λ retrieves the odds for each case:

$$\frac{p}{1-p} = e^{b_0+b_1x_1+b_2x_2+\cdots b_nx_n}, \qquad 0 < p < 1 \qquad \text{(S2)}$$

where e is Napier's number. The probability p can then be expressed as follows:

$$p = \frac{e^{b_0+b_1x_1+b_2x_2+\cdots b_nx_n}}{1 + e^{b_0+b_1x_1+b_2x_2+\cdots b_nx_n}}, \qquad 0 < p < 1 \qquad \text{(S3)}$$

To generate our model, we calculate each b_i, where i∈[1…n] for each variable x_i where i∈[1…n]. For each predictor (i.e., symptom) x_i, the odds ratio calculated by logistic regression (i.e. the exponent of b_i) is used as a corresponding weight in a subsequent multiple correspondence analysis. Finally, the probability p is used to score each case.

Decision Trees via Quest

QUEST was originally proposed by Loh and Shih [22]. Let us consider cluster (i.e. phenotype) membership as a dependent nominal variable Y with J classes (equal to the number of phenotypes), and each symptom as a categorical (nominal) predictor X.

The decision tree expands by testing for the best predictors among the input,

For each categorical predictor X, QUEST performs a chi-square test of independence at each node n, subsequently calculating the corresponding p-value:

$$p_x = \Pr(\chi_d^2 > X^2) \qquad \text{(S4)}$$

where d represents the degrees of freedom for χ_d^2, for a predictor X with K_n categories:

$$d = (J_n - 1)(K_n - 1) \qquad \text{(S5)}$$

The growth procedure depends on establishing the best splitting predictor at each node based on the smallest p-value. Conversely, the "stopping" process is determined by several stopping criteria. In our study, the applied criterion was node purity, i.e. the complete separation of a predictor variable on a dependent variable class.
